# A naturally occurring variant of *MBD4* causes maternal germline hypermutation in primates

**DOI:** 10.1101/gr.277977.123

**Published:** 2023-12

**Authors:** Alexandra M. Stendahl, Rashesh Sanghvi, Samuel Peterson, Karina Ray, Ana C. Lima, Raheleh Rahbari, Donald F. Conrad

**Affiliations:** 1 Division of Genetics, Oregon National Primate Research Center, Beaverton, Oregon 97006, USA;; 2 Cancer, Ageing and Somatic Mutation (CASM), Wellcome Trust Sanger Institute, Hinxton, Cambridge CB10 1SA, United Kingdom

## Abstract

As part of an ongoing genome sequencing project at the Oregon National Primate Research Center, we identified a rhesus macaque with a rare homozygous frameshift mutation in the gene methyl-CpG binding domain 4, DNA glycosylase (*MBD4*). MBD4 is responsible for the repair of C > T deamination mutations at CpG dinucleotides and has been linked to somatic hypermutation and cancer predisposition in humans. We show here that MBD4-associated hypermutation also affects the germline: The six offspring of the *MBD4*-null dam have a fourfold to sixfold increase in de novo mutation burden. This excess burden was predominantly C > T mutations at CpG dinucleotides consistent with *MBD4* loss of function in the dam. There was also a significant excess of C > T at CpA sites, indicating an important, unappreciated role for MBD4 to repair deamination in CpA contexts. The *MBD4*-null dam developed sustained eosinophilia later in life, but we saw no other signs of neoplastic processes associated with *MBD4* loss of function in humans nor any obvious disease in the hypermutated offspring. This work provides the first evidence for a genetic factor causing hypermutation in the maternal germline of a mammal and adds to the very small list of naturally occurring variants known to modulate germline mutation rates in mammals.

The methyl-CpG binding domain 4, DNA glycosylase (MBD4) repairs C > T mutations through the base excision repair pathway by removing the thymine in a G–T mismatch (Pidugu et al. 2021). MBD4 is specifically active at repairing the spontaneous deamination of 5-methyl-cytosine to thymine, one of the most common somatic mutations in the genome (Sanders et al. 2018).

MBD4 is critical for genome stability and preventing mutations, and loss of function of *MBD4* has recently been associated with a higher risk of MBD4-associated neoplasia syndrome, a multiorgan tumor predisposition syndrome (Palles et al. 2022). It is further associated with several specific cancers, including adenomatous colorectal polyposis, acute myeloid leukemia, and uveal melanoma (Millar et al. 2002; Derrien et al. 2021; Palles et al. 2022). Additionally, many types of cancerous tumors are often found to contain mutations in the *MBD4* gene, especially gastric, endometrial, and pancreatic carcinomas (Wong et al. 2002). Congruent with the function of *MBD4*, C > T mutations are found in high incidence in gastrointestinal tumors of *MBD4*^−*/*−^ mice, with most occurring at CpG dinucleotides (Wong et al. 2002).

As part of an ongoing genetic study at Oregon National Primate Research Center (ONPRC), we identified a rhesus macaque (ID 26537) with a germline homozygous TC > C deletion in the *MBD4* gene at 2:147,059,371 (Mmul_10 genome assembly) (Fig. 1). In this short report, we describe a germline hypermutation phenotype that we ascribe to this variant, and we analyze the patterns of de novo mutations (DNMs) across six offspring of 26537 to generate hypotheses about the timing and molecular basis for this effect.

**Figure 1.**
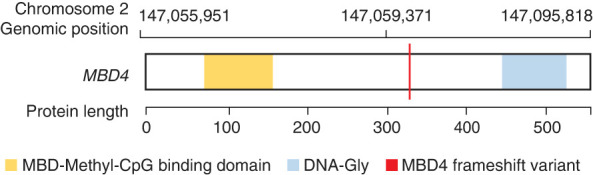
Identification of a biallelic frameshift mutation in *MBD4* in 26537. Location of the mutation with respect to genome (Mmul_10) and protein (ENSMMUG00000012723) coordinates.

## Results

Sequencing of the parents of 26537 confirmed that the *MBD4* genotype is an inherited homozygous deletion (Supplemental Fig. S1). In macaques, the MBD4 protein is 572 amino acids long and consists of a methyl binding domain at the N terminus and a glycosylase domain at the C terminus, which is involved in the DNA repair. The mutation in 26537 results in a frameshift (ENSMMUG00000012723:c.984delC,p.Gly329fs), leading to an isoleucine-to-serine substitution at position 330 (Ile330Ser) and an early truncation at the following amino acid (Ile331AUU). Monkey 26537 is the only known homozygote at ONPRC or in mGAP (https://mgap.ohsu.edu), the database of macaque genomes (currently 3455 monkeys) (Bimber et al. 2019). The allele frequency in mGAP is 0.0072. mGAP contains genotype data on macaques from over 10 centers around the United States. Although 41% of the individuals in mGAP are from ONPRC, 38/39 *MBD4* heterozygotes were found in ONPRC animals, the one exception being an animal from Rocky Mountain Laboratories.

**Figure 2.**
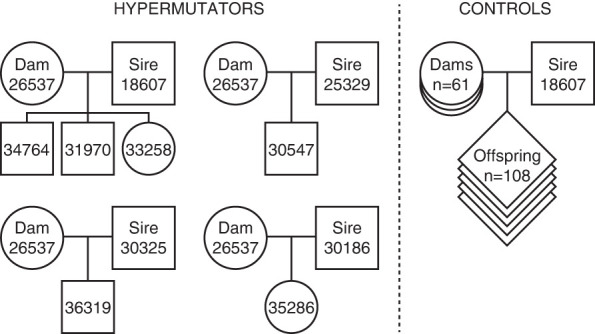
Overview of families sequenced for the study.

26537 was an Indian-origin rhesus macaque born at ONPRC in 2007. She gave birth to six offspring between the ages of four and 10 and was euthanized at age 14 based on her participation in an experimental protocol unrelated to this study. Through an ongoing genome sequencing study, we sequenced 26537's six offspring and the sires of her offspring to obtain complete trios (Fig. 2; Supplemental Table S1). We also sequenced a large pedigree of macaques from a prolific male breeder (ID: 18607), including his 111 offspring and the 61 dams (Supplemental Table S1). Three of 26537's offspring were sired by this prolific male breeder. Three of 18607's offspring were produced from matings with heterozygous carriers of the *MBD4* mutation (18210 and 18147), providing a limited opportunity to assess potential dominant or additive effects of the mutation.

In our analysis of the prolific male's pedigree, all of 26537's offspring were flagged owing to the high number of DNMs. Germline DNMs are mutations that appear in an offspring without detection in the somatic cells of either parent. Humans have a germline mutation rate of 1.2 × 10^−8^ to 1.3 × 10^−8^ per position per generation (about 60–70 DNMs), whereas macaques appear to have a mutation rate of 0.58 × 10^−8^ to 0.77 × 10^−8^ per site per generation (Conrad et al. 2011; Kong et al. 2012; Rahbari et al. 2016; Wang et al. 2020; Bergeron et al. 2021). Germline DNMs can originate as errors in DNA replication during gametogenesis or other processes. Males tend to pass on more DNMs to their offspring, which may be a result of the continuous germ cell division in spermatogenesis as opposed to oogenesis in females, which is completed before birth (Crow 2000). However, as Abascal et al. (2021) found that somatic mutation rate is more related to cell type than number of divisions, there may be additional mutational processes involved in DNMs in sperm. Older males are especially at risk of passing high numbers of mutations to their offspring, contributing approximately 1.5 to two mutations per year of age (Kong et al. 2012; Rahbari et al. 2016; Abascal et al. 2021; Moore et al. 2021). Although some studies do show a maternal age effect, it is considerably smaller, approximately 0.3 to 0.5 mutations per year (Kong et al. 2012; Wong et al. 2016; Jónsson et al. 2017).

**Figure 3.**
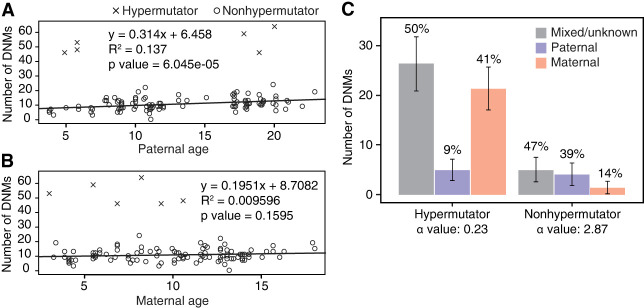
Children of 26537 show rates of germline de novo mutation (DNM) four- to sixfold higher than children from control parents. (*A*) Relationship between paternal age and number of DNMs observed in offspring. (*B*) Relationship between maternal age and number of DNMs observed in offspring. The observed paternal and maternal age effects seen in non-26537 offspring largely adhere to previous literature (Kong et al. 2012; Rahbari et al. 2016; Wang et al. 2020). Best fit lines and numerical values within each panel correspond to a standard linear model fit to control (nonhypermutator) data only. (*C*) Percentage of DNMs observed on paternally and maternally inherited chromosomes. Read-based phasing of DNMs shows an excess of maternally derived DNMs in offspring of 26537 not observed in control offspring. The alpha estimate for each set of phased mutations is shown. (Alpha) Ratio of paternal:maternal mutations.

In this study, we sequenced the genomes of 176 animals to a mean coverage of 23.6×. Adjusting for callable bases, our filtered DNM callset from the control trios produced a mutation rate estimate of 0.81 × 10^−8^ per base pair per generation. Although the average rhesus macaque carried 10.5 DNMs (stdev = 4), offspring of 26537 carried 46–64 DNMs (Supplemental Table S1). To formally evaluate the impact of the *MBD4* mutation, we modeled the mutation burden in all sequenced individuals, as well as separate terms for *MBD4* heterozygotes and homozygotes (Methods). We found significant terms for paternal age and *MBD4* homozygosity but not for maternal age or *MBD4* heterozygote status (Fig. 3A,B). The number of callable bases per genome, which we use as a proxy for power, was not significantly associated with mutation burden. There are only two heterozygous dams in our analysis, with three offspring between the two of them, so power was limited to see an effect of a single copy of the *MBD4* frameshift allele on germline mutation.

Despite being sired by four different males, all six offspring of 26537 had extremely high numbers of C > T mutations at CpG locations (between 25 and 39) compared with the average of one mutation per monkey not birthed by 26537. Because MBD4 repairs C > T mutations at CpG locations, an enrichment in C > T mutations at CpG locations originating from the maternal haplotype is consistent with the *MBD4* mutation. Moreover, read-based phasing revealed a 16- to 23-fold increase in maternally derived mutations in the offspring of 26537 compared with control trios (average of 21.33 maternal mutations vs. 1.43, *P* = 1 × 10^−95^ Poisson regression) (Fig. 3C; Supplemental Table S2), whereas there was no difference in paternally derived mutations (5.0 vs. 4.1, *P* = 0.176).

**Figure 4.**
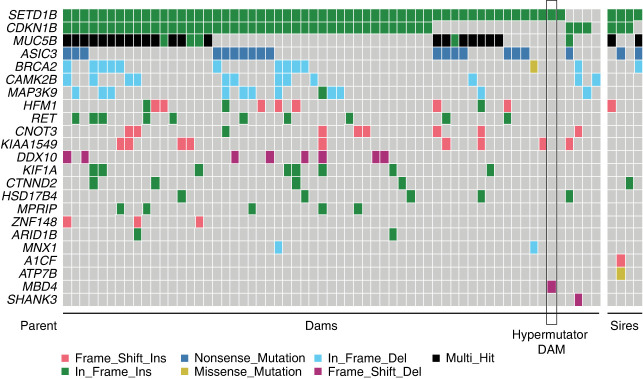
*MBD4* is the only mutated DNA repair gene private to 26537. To confirm *MBD4* is the most likely candidate for the hypermutation phenotype, we searched for biallelic damaging mutations across 1381 candidate genes for germline hypermutation derived from the literature (Supplemental Table S3). We identified candidate mutations in 23 genes, shown here. *MBD4* was the only gene specifically mutated in 26537 and not other parents in the study.

To build further evidence implicating *MBD4* loss of function as the cause of the observed hypermutation, we screened 26537 for other potentially causal mutations in genes related to genome instability genes derived from literature (Methods; Supplemental Tables S3–S6), assessing all other parents included in our study to provide context. Although 23 genes were mutated in the cohort, the only uniquely mutated gene in 26537 was *MBD4* (Fig. 4).

**Figure 5.**
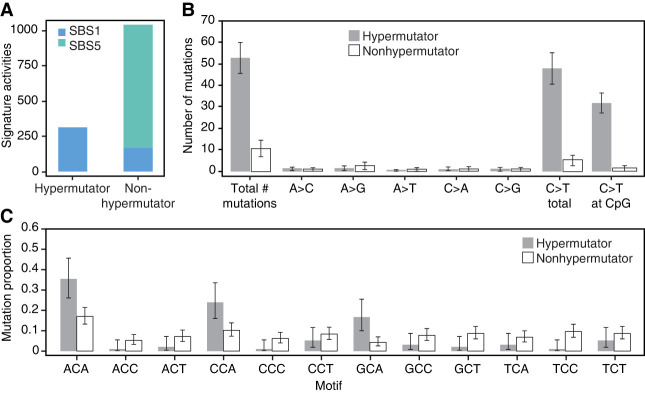
Mutation signature analysis of *MBD4* germline mutations. (*A*) Unlike germline mutational signatures in control individuals, which are mostly signature 5 with a smaller proportion of signature 1, the majority of mutations in the hypermutated offspring were attributed to signature 1 with very small contribution from signature 5. (*B*) Of the six possible substitution types, C > T was the only based substitution with a significant excess in offspring of 26537 (hypermutator) compared with offspring from the control families (nonhypermutator). Most of these C > T changes were in a CpG context, consistent with the known function of MBD4 in repairing spontaneous methyl-cytosine deamination. (*C*) The remaining excess of C > T changes were observed at CpA positions (*P* = 3 × 10^−4^ for contrast of hypermutator vs. control by Poisson regression).

Mutational signature analysis (Tate et al. 2019) of all 26537 offspring showed almost all the mutations were attributed to signature 1, whereas in the control trios, only 20% of mutations were attributed to signature 1 and the rest (80%) were signature 5 (Fig. 5A). This composition of mutation signatures is consistent with the mutation data in humans: Somatic mutations from *MBD4*^−/−^ individuals have an excess of signature 1 (Palles et al. 2022), whereas germline mutations in typical humans are a mixture of signatures 1 and 5 (Moore et al. 2021). Mutation signature 1 has been attributed to cytosine deamination and is primarily composed of C > T mutations. Indeed, the vast majority of mutational excess observed in the offspring of 26537 were C > T mutations (Fig. 5B). Although most cytosine deamination is thought to occur at CpGs, ∼40% of the C > T mutations in the hypermutator offspring were not in CpG. We used Poisson regression to test for enrichment of C > T mutations across trinucleotide contexts and observed a clear enrichment in three trinucleotides that did not contain CpG: ACA, CCA, GCA (Fig. 5C). This indicates a clear pattern of C > T mutations in locations where a cytosine is followed by an adenine (CpA). We fit a generalized linear model to the C > T mutation counts in CpA across all trios, including *MBD4* genotype and parental ages as covariates; the *MBD4*^−/−^ genotype was significantly associated (*P* = 3.5 × 10^−4^) but not *MBD4*^+/−^ (*P* = 0.69). Although CpG methylation is maintained in all tissues over the life span of an animal, CpA methylation is observed primarily in fetal development and in adult neurons (Greenberg and Bourc'his 2019).

To understand whether the hypermutation phenotype or *MBD4* frameshift mutation had an effect on offspring or dam health, we reviewed electronic health records and necropsy information if present for these individuals. None of the hypermutated offspring appear to have any major health concerns. While she was alive, 26537 did not appear to show any major health issues, aside from a single stillbirth; however, tissue from the stillborn was not available for analysis. 26537 was necropsied at 14 yr of age as part of another research protocol at ONPRC. The timing of the necropsy was based on the overall design of that study and did not reflect any health or fertility issues of 26537. There were no gross indications of neoplasia at necropsy nor during histological analysis of brain, gastric tissues, or lymph nodes.

**Figure 6.**
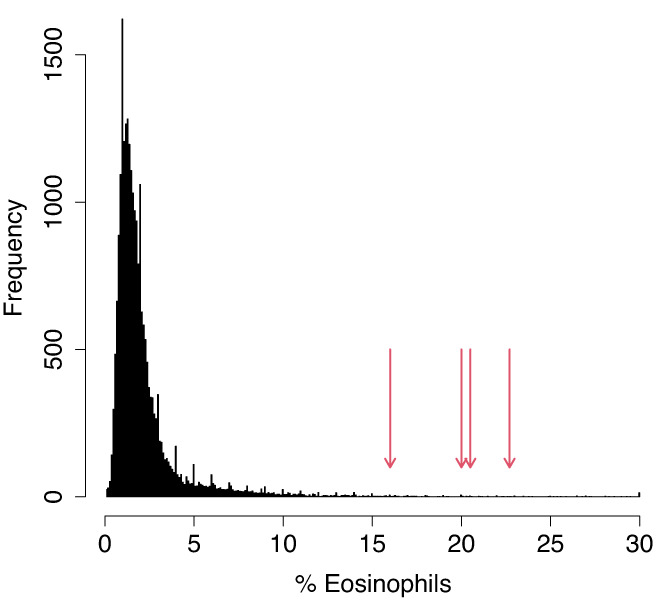
Rhesus macaque 26537 showed sustained eosinophilia at age 14. Four independent CBCs run over several weeks showed 16%–22.7% eosinophils (red arrows). These numbers were in the top 0.5% of more than 25,000 CBCs run at ONPRC since 2016 (histogram).

At periodic intervals as part of their wellness check-up, monkeys at ONPRC receive complete blood counts (CBCs) and complete metabolic panels. Four CBCs run on 26537 during the last year of her life revealed sustained eosinophilia, with an average measurement of 19.8% eosinophils (all other measures of the CBC and CMP were in the normal range during this time). We compared these high eosinophilic measures to 24,638 CBCs run on 4699 unique animals at ONPRC since 2016. The average percentage of EOS for animals tested over this time was 2.2%; only 71 tests (0.3%) reported higher EOS counts (Fig. 6). Fecal parasitology excluded parasitemia as a possible cause for eosinophilia in 26537. Eosinophilia is sometimes observed in several cancers and cancer-related syndromes, especially eosinophilic leukemia and myelodysplastic syndrome (MDS). Both MDS and acute myeloid leukemia have been observed in humans with biallelic mutation of *MBD4* (Palles et al. 2022). These findings provide evidence that the *MBD4* mutations in 26537 potentially had functional consequences at a cellular level, beyond deficiencies in DNA repair.

## Discussion

Through a large genome sequencing project at ONPRC, we identified six hypermutated monkeys, all originating from a dam with a homozygous mutation in the *MBD4* gene causing a premature stop codon. This discovery adds to the short list of naturally occurring genetic variants that have been found to modulate germline mutation rates, in humans, in mice, and now in rhesus macaques.

Hypermutation can be the result of both environmental factors, such as parental exposure to chemotherapy, and genetic factors, such as a knockout of a DNA repair gene. Germline hypermutation (a large excess of DNMs) is quite rare in humans. Kaplanis et al. (2022) found 12 individuals out of 21,879 human families with a hypermutated phenotype; only two of these could be attributed to genetic causes. Both cases were born to fathers with a germline hypermutation phenotype attributed to rare biallelic mutations in the DNA repair genes *XPC* and *MPG*. *MPG* encodes *N*-methylpurine DNA glycosylase, which, similar to MBD4, is involved in correction of deaminated purines by the base-excision repair pathway. Inherited variation in another DNA glycosylase, *MUTYH*, can increase somatic mutation rates in humans (Robinson et al. 2022) and in the germline in mice (Sasani et al. 2022). Notably, our finding of a maternal germline hypermutator phenotype appears to be the first clear evidence of genetic variants affecting germline mutation in a female mammal. It seems plausible that, as the male germline is subject to extensive methylation and as *MBD4* is strongly expressed in male germ cells (Mahyari et al. 2023), zygotic *MBD4* loss of function in a male would also lead to an increased rate of germline mutation. Indeed, all of the paternal hypermutator alleles reported to date affect genes that show robust expression in human oocytes (Menezo et al. 2007) and, as such, would be expected to lead to hypermutation of the female germline as well. Because the baseline mutation rate in male primates (including humans) is much higher than in females, we expect that statistical power will continue to favor ascertainment of germline hypermutators from affected males when studying population samples, even for genetic variants that affect male and female germlines equally.

Many questions remain about how, as well as when during the lifecycle, the germline is vulnerable to DNA damage. The data presented here may hold some important clues regarding the timing of cytosine deamination and its repair in the maternal germline. The DNMs transmitted from 26537 to her offspring were present in the oocytes that generated each offspring. All offspring from 26537 were heterozygous for the *MBD4* mutation, meaning that they were formed by fertilization of an *MBD4*^−*/*−^ oocyte by a *MBD4*^*+/+*^ spermatozoa. In principle, the mutations in the *MBD4*^−*/*−^ oocytes could have originated at any time point in the lifecycle of 26537, from the zygote to the fertilization of each *MBD4*^−*/*−^ oocyte. Multiple observations in this study would seem to restrict the plausible developmental window during which the hypermutator phenotype was active. First, there is no detectable effect of maternal age on the number of mutations in the six offspring of 26537 (Fig. 3B). A Poisson regression model fit to the mutation burden in hypermutator trios alone found a slightly negative but not significant slope (beta_age_ = −0.02, *P* = 0.55), and a model fit to the full data found no interaction between hypermutator status and age (beta_age*hypermutator_status_ = −1.13, *P* = 0.21). Because of the small number of offspring, we cannot exclude the possibility that there is a small age-related accumulation of mutation over time, but a clock-like steady accumulation of mutation throughout the life of 26537 cannot be the primary source of the excess DNM. Second, there is minimal sharing of mutations among the offspring of 26537: Four of 316 (1.2%) filtered mutations in the offspring were observed more than once (Supplemental Table S7). This suggests that none of the mutations were present in the embryonic progenitor of all germ cells, and very few of the 316 mutations were present in the embryo at the time of the PGC specification. Finally, in addition to the expected excess of CpG mutations in the offspring of 26537, we observed a secondary enrichment in CpA contexts, accounting for 28% of all calls in these children (Fig. 5C). Non-CpG methylation, especially involving CpA, occurs genome-wide in oocytes of mice (Shirane et al. 2013) and humans (Yu et al. 2017). Human oocytes show dramatic (>100%) increases in CpA methylation during oocyte maturation, whereas CpG methylation levels stay stable (Yu et al. 2017). Furthermore, *MBD4* is expressed in developing oocytes, and its expression is highly correlated to the oocyte expression levels of *DNMT3A*, a methyltransferase responsible for non-CpG methylation. Both *MBD4* and *DNMT3A* show a remarkable increase in transcription in human fetal ovary during 16-wk gestation, when oocytes are first reaching the germinal vesicle stage (Galetzka et al. 2007). Taken together, we propose that the source of the germline hypermutation phenotype from *MBD4*^−/−^ animals is not owing to a loss of “maintenance” deamination repair that accumulates over the lifetime of the animal but instead is owing to a time-restricted window of damage, perhaps induced during de novo CpA methylation.

## Methods

### Genome sequencing and primary analysis

DNA was extracted from peripheral blood. Whole-genome sequencing libraries were constructed using a PCR-free method (Illumina). Libraries were assessed for quality and quantity by real-time PCR and Tapestation (Agilent). Libraries were normalized and mixed and then sequenced with a 2 × 150-bp paired-end protocol on S4 flow cells using a NovaSeq 6000. Basecall files were converted to FASTQ files using bcl2fastq (Illumina). Reads were aligned to Mmul_10 using BWA-MEM v.0.7.17-r1188 (Li 2013). The average mapped read depth of all genomes in the study was 23.6× (minimum 14.5×, maximum 35.8×) (Supplemental Table S1). Mapping of sample IDs used in this study to NCBI Sequence Read Archive (SRA; https://www.ncbi.nlm.nih.gov/sra) accessions and mGAP IDs is provided in Supplemental Table S8.

### DNM identification and filtering

Initial candidate DNMs were identified using DeNovoGear (Ramu et al. 2013). We first selected sites with a probability of de novo single-base-pair mutation greater than 0.5. With this filter, DeNovoGear identified an average of 6144 candidate mutations per individual (for exact counts of mutations identified and filtered in each sample, see Supplemental Table S1; for exact filtering commands, see Supplemental Code). These sites were further filtered to include sites where offspring and parent read depth was between 4 and 80 reads, which removed an average of 3848 mutations. Further filtering included requiring offspring to have a variant allele frequency (VAF) between 35% and 70% for mutations on autosomes. Mutations on the X Chromosome should have a VAF between 35% and 70% for female offspring and >70% for male offspring. Mutations on the Y Chromosome were excluded. Alternate allele reads must appear on both forward and reverse strands, with at least two reads supporting the alternate allele. This filtering eliminated an additional 888 mutations on average. We removed sites that fell in highly repetitive regions (UCSC simple repeats track) and in known segmental duplications (UCSC Segmental Duplication track) (Haeussler et al. 2019), which removed an average of 50 and 681 mutations, respectively. We removed mutations that were relatively common in the macaque population, as defined by having an allele frequency in mGAP greater than 0.01, which removed 587 mutations. We removed variants in which either parent had more than one alternate allele read or >10% VAF, which removed 77 mutations on average. Finally, we used a binomial test with FDR correction (adjusted *P*-value < 0.05) to settle on our final set of DNMs, which removed between zero and three mutations.

### Phasing de novo mutations

To confirm the hypermutation phenotype was a result of mutations coming from 26537, we phased mutations to either the maternal or paternal allele. Germline mutations were jointly called for each trio using HaplotypeCaller (Poplin et al. 2018), and the DNMs were phased using PhaseMyDeNovo using default parameters (https://github.com/queenjobo/PhaseMyDeNovo). We were able to phase 50% of mutations to a single parent in hypermutators and 53% of mutations in nonhypermutators. Nonhypermutated animals had 74% of phaseable mutations phased to the paternal side, whereas the hypermutated offspring had 81% of phaseable mutations phased to the maternal side. The paternal-to-maternal ratio (alpha-value = 2.87) of the nonhypermutators was similar to that reported in literature (about three) (Wang et al. 2020) compared with 0.23 for hypermutators.

### Mutation signature extraction

To determine the mutational processes resulting in the hypermutation in the six offspring of 26537, we compared the DNM profiles by extracting mutational signatures using SigProfilerExtractor (v 1.1.7) and estimating their contribution to the mutation burden (Islam et al. 2022). To accurately estimate signature proportions reflective of the rhesus genome, we renormalized the COSMIC v3.2 SBS human signature table based on the GRCh37 assembly to reflect the trinucleotide frequencies present in Mmul_10. Mutation signatures, as statistical summaries of context-dependent mutation, rely on the base composition of euchromatic DNA. The base composition of euchromatic DNA in GRCh37 is 99.9%, the same as the newer GRCh38 assembly and subsequent releases; the other assembly differences involve changes to heterochromatic DNA (which we do not analyze here), a small amount of new sequence in assembly gaps, and the addition of small, alternate haplotypes at 178 loci, also not considered here (Guo et al. 2017). Thus, an analysis based on a newer assembly should produce nearly identical results.

### Linear modeling of mutation burden

We used Poisson regression to model the effects of maternal age, paternal age, *MBD4* genotype, and sequencing coverage on observed mutation counts. To account for differences in sequencing depth among trios, we calculated the number of callable bases for each sample using mosdepth (Pedersen and Quinlan 2018) with the categories “NO_COVERAGE,” “LOW_COVERAGE,” “CALLABLE,” and “HIGH_COVERAGE” split as “‐‐quantize 0:1:4:80:.” The average number of callable bases per genome was 1.32 Gb. We find this number a reasonable proxy for power because normalizing the total number of mutations to the callable genome size produces a mutation rate estimate very similar to published values for macaque. We fit this model separately for the total number of mutations, as well as labeled mutation subsets: A > C, A > T, A > G, C > A, C > G, C > T, C > T in the CpG context; C > A in the CpG context; and C > T in the non-CpG context. To test for an effect of hypermutator status on the age-related accumulation of mutations, we fit a version of this model with an additional interaction term between hypermutator status and age.

### Mapping a genetic cause of hypermutation in 26537

To identify a genetic cause for the hypermutation phenotype of 26537, we used two approaches. First, we used mGAP, the macaque genotype and phenotype database, to annotate allele frequencies for all variants in the joint VCF of individuals in the study. We then selected all coding variants from 26537 with mGAP minor allele frequency < 1%, homozygous in 26537, and not homozygous in the other trio parents. Sequencing alignments and gene annotations were then used to curate the resulting list, eliminating sequencing errors and annotation errors. Of the remaining variants, the only variant that affected a gene with a known function relating to DNA repair was *MBD4*.

Second, to confirm MBD4 as the most likely cause of the hypermutation phenotype, we carefully investigated 1378 candidate hypermutation genes (Supplemental Tables S3–S6), chosen based on previous literature tying them to genome instability. These are cancer predisposition genes in pediatric cancer (Zhang et al. 2015), DNA damage repair genes from The Cancer Genome Atlas (Knijnenburg et al. 2018), an in-house cancer driver list (“Tarpey driver genes”), cancer gene consensus genes from COSMIC (https://cancer.sanger.ac.uk/census), and genes identified as involved in developmental disorders (“DDD_genes”; https://www.deciphergenomics.org/ddd/ddgenes). The full list of genes interrogated is provided in Supplemental Table S3. The joint VCF of germline genotypes was annotated using VEP. We then extracted all mutations in these genes that were annotated by VEP as “high” or “moderate” impact, and identified gene candidates with biallelic damaging mutations in at least one dam or sire, which generated a short list of 23 genes (Fig. 4).

## Data access

The whole-genome sequencing data generated in this study have been submitted to the NCBI BioProject database (https://www.ncbi.nlm.nih.gov/bioproject/) under accession number PRJNA382404. The code and input data to reproduce the analyses and figures described here are available from GitHub (https://github.com/conradlab/StendahlEtAl) and as Supplemental Code.

## Supplementary Material

Supplement 1

Supplement 2

Supplement 3
