# Supplementary material for: A naturally occurring variant of MBD4 causes maternal germline hypermutation in primates
Source: Genome Res. 2023 Dec;33(12):2053–9. doi: 10.1101/gr.277977.123 (PMC10760519; doi:10.1101/gr.277977.123)
Supplement: Supplement 3 [file Supplemental__Figure_S1.pdf]

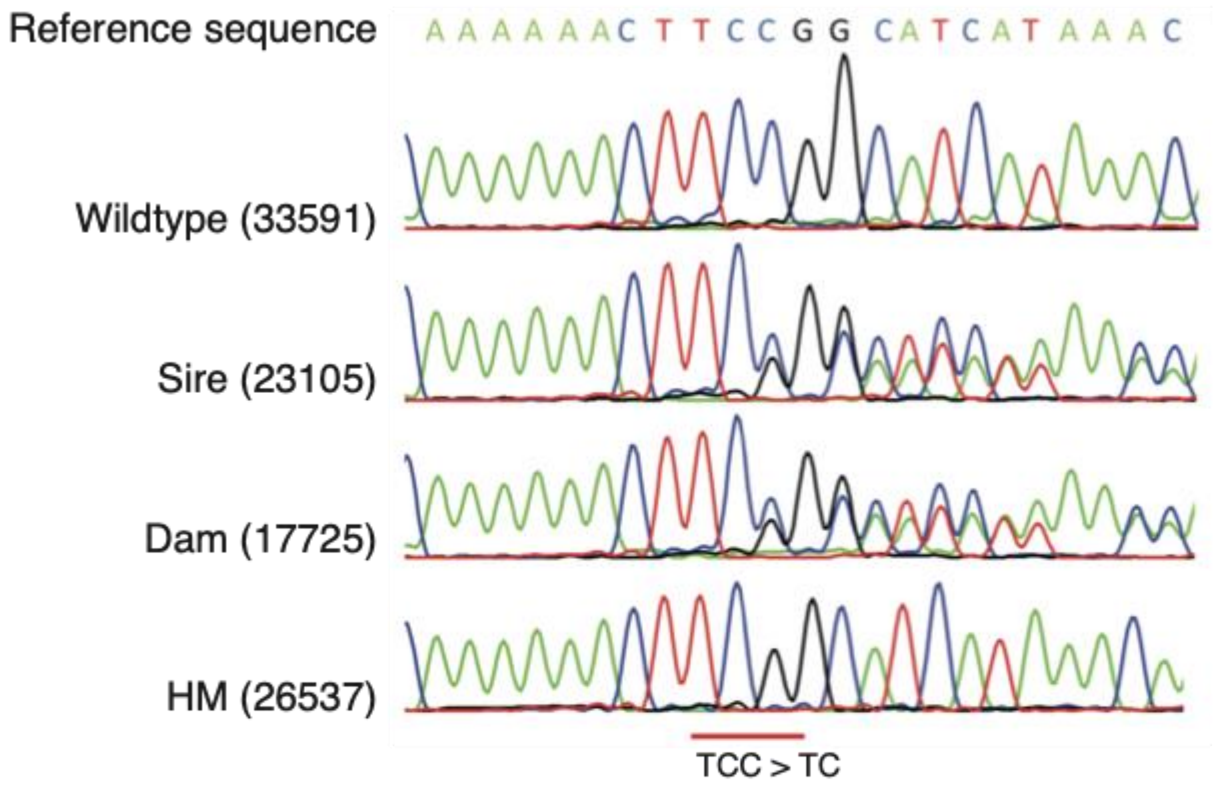

Figure S1. Sanger Validation of mutation. Sanger validation of the frameshift indicates germline inheritance from both parents of 26537. HM= hypermutator. Sire and Dam are parents of 26537.
